# Development of a bead-based Luminex assay using lipopolysaccharide specific monoclonal antibodies to detect biological threats from *Brucella species*

**DOI:** 10.1186/s12866-015-0534-1

**Published:** 2015-10-05

**Authors:** Angelika Silbereisen, Marco Tamborrini, Matthias Wittwer, Nadia Schürch, Gerd Pluschke

**Affiliations:** Department of Medical Parasitology and Infection Biology, Swiss Tropical and Public Health Institute, Basel, Switzerland; University of Basel, Basel, Switzerland; Federal Office for Civil Protection, Spiez Laboratory, Spiez, Switzerland

**Keywords:** Brucellosis, Luminex, Antigen capture assay, Monoclonal antibodies, Multiplex

## Abstract

**Background:**

*Brucella*, a Gram-negative bacterium, is classified as a potential bioterrorism agent mainly due to the low dose needed to cause infection and the ability to transmit the bacteria via aerosols. Goats/sheep, cattle, pigs, dogs, sheep and rodents are infected by *B. melitensis, B. abortus, B. suis, B. canis, B. ovis* and *B. neotomae*, respectively, the six classical *Brucella* species. Most human cases are caused by *B. melitensis* and *B. abortus*. Our aim was to specifically detect *Brucellae* with ‘smooth’ lipopolysaccharide (LPS) using a highly sensitive monoclonal antibody (mAb) based immunological assay.

**Methods:**

To complement molecular detection systems for potential bioterror agents, as required by international biodefense regulations, sets of mAbs were generated by B cell hybridoma technology and used to develop immunological assays. The combination of mAbs most suitable for an antigen capture assay format was identified and an immunoassay using the Luminex xMAP technology was developed.

**Results:**

MAbs specific for the LPS O-antigen of *Brucella spp*. were generated by immunising mice with inactivated *B. melitensis* or *B. abortus* cells. Most mAbs recognised both *B. melitensis* and *B. abortus* and antigen binding was not impeded by inactivation of the bacterial cells by γ irradiation, formalin or heat treatment, a step required to analyse the samples immunologically under biosafety level two conditions. The Luminex assay recognised all tested *Brucella* species with ‘smooth’ LPS with detection limits of 2 × 10^2^ to 8 × 10^4^ cells per mL, depending on the species tested. Milk samples spiked with *Brucella spp.* cells were identified successfully using the Luminex assay. In addition, the bead-based immunoassay was integrated into a multiplex format, allowing for simultaneous, rapid and specific detection of *Brucella spp., Bacillus anthracis*, *Francisella tularensis* and *Yersinia pestis* within a single sample.

**Conclusion:**

Overall, the robust Luminex assay should allow detection of *Brucella spp.* in both natural outbreak and bio-threat situations.

**Electronic supplementary material:**

The online version of this article (doi:10.1186/s12866-015-0534-1) contains supplementary material, which is available to authorized users.

## Background

Brucellosis, a zoonotic bacterial disease caused by Gram-negative *Brucellae* and classified as a potential bioterrorism disease [1], leads to abortions in animals and flu-like symptoms with periodic bouts of fever in humans. *B. melitensis, B. abortus, B. suis, B. canis, B. ovis* and *B. neotomae* are the six classical species that infect mainly goats/sheep, cattle, pigs, dogs, sheep and rodents, respectively, while *B. melitensis* and *B. abortus* cause most of the human infections [2–4]. Like other Gram-negative bacteria, *Brucellae* express lipopolysaccharide (LPS), a major component of the outer membrane. The three structural components of LPS are the lipid A, the core oligosaccharide and the O-polysaccharide (O-antigen). In ‘smooth’ *Brucella* species, the O-polysaccharide is a linear polymer of 4,6-dideoxy-4-formamido-α-D-mannopyranosyl residues, whereas ‘rough’ strains have a truncated version without the O-antigen [5, 6]. *Brucella* LPS is able to induce protective antibodies [7–9], which are potentially important for serological diagnosis [10–16]. Because of the threat posed by natural outbreaks or by a deliberate release of the bacteria as a bioterror agent [17], there is a need for rapid and reliable identification systems, preferably based on multiplex formats covering a range of relevant species. This is especially important for fastidious agents such as *Brucella* or *Francisella* species where tracing by cultivation is hampered by long cultivation time.

The aim of this study was to develop a rapid and sensitive immunological assay to detect all *Brucellae* with ‘smooth’ LPS, particularly *B. melitensis* and *B. abortus*. To this end, monoclonal antibodies (mAbs) specific for *Brucella* LPS were generated and used to design a highly specific and sensitive antigen capture assay. An optimal combination of mAbs was identified and a *Brucella* LPS specific Luminex xMAP assay [18, 19] was developed, capable of detecting four of the major *Brucella* species (*B. melitensis, B. abortus, B. suis, B. neotomae*) with high sensitivity. Additionally, the Luminex assay works in a multiplex format, simultaneously detecting four category A and B bacterial bioterrorism agents and is suitable for detecting *Brucella* in complex samples.

## Methods

### Ethics statement

This study was carried out in strict accordance with the Rules and Regulations for the Protection of Animal Rights (Tierschutzverordnung) of the Swiss Federal Food Safety and Veterinary Office. The protocol was granted ethical approval by the Veterinary Office of the county of Basel-Stadt, Switzerland (Permit Number: 2375).

### Production and inactivation of bacteria

Bacterial strains used in this study are listed in Table 1.

**Table 1:** Bacterial strains

Bacterial species	Strain	Inactivation method	Source
*Brucella spp. (biotype)*			
* B. abortus (1)*	NCTC 10093 544	γ, formalin, heat	SL
	19S	formalin	SL
	A146-10	formalin	RKI
* B. abortus (3)*	A104-10 Uckermark	γ	RKI
* B. canis*	NCTC 10854 RM-666	formalin	SL
* B. ovis*	CNCTC 6741	heat	RKI
* B. melitensis (1)*	NCTC 10094 16 M	γ, formalin, heat	SL
	ICM 3.33	formalin	SL
	ICM 583/2003	formalin	SL
	ICM 91/2004	formalin	SL
	102A01C2F	formalin	SL
	A146-13	formalin	RKI
	A104-11 Tgb. Nr. 117518	γ	RKI
* B. melitensis (2)*	A104-12 799/97, B3898	γ	RKI
* B. melitensis (3)*	A104-13 210739, Mainz	γ	RKI
* B. suis (1)*	NCTC 10316 1330	formalin	SL
* B. suis (2)*	A 104–14 Rostock	heat	RKI
* B. neotomae*	A148-7 5 K33	γ	RKI
Other bacteria			
* F. tularensis tularensis*	ATCC 6223	formalin	SL
* F. tularensis holarctica*	LVS, NCTC 10857	formalin	SL
* B. anthracis (spores)*	Böhm 73202.2000 (PX02+)	formalin	SL
	Böhm A1 (PX01+)	formalin	SL
* S. typhimurium*	ATCC 14028	formalin	SL
* Y. enterocolitica*	310 (IT2, ST9) O9	formalin	SL
* Y. pestis*	CO92	γ, formalin	SL
* O. anthropi*	ATCC 49188	formalin	SL
* E. coli*	O:157, 15326	formalin	SL
* B. mallei*	NCTC 03709 106	formalin	SL
* B. pseudomallei*	H05410-0490	formalin	SL
* V. cholera*	O1, ATCC 14734	formalin	SL

Bacteria were inactivated by 3 % formalin, heat (60 °C for >20 h) or gamma (γ) irradiation (30–40 kGy). SL = Spiez Laboratory (Federal Office for Civil Protection, Spiez, Switzerland). RKI = Robert Koch Institute (Berlin, Germany)

*Brucella spp.* were cultured on Columbia blood agar plates supplemented with 5 % goat blood [20]. Bacteria were inactivated by 3 % formalin (55 °C for 15 min), heat (60 °C for >20 h) or gamma (γ) irradiation at 30–40 kGy (Leoni Studer Hard AG, Däniken, Switzerland). Sterility was checked by incubating bacteria for three days on agar plates and no growth was observed.

### Production of anti-LPS mAbs

To produce *Brucella* LPS-specific mAbs, mice carrying human immunoglobulin Cγ1 heavy and Cκ light chain gene segments [21] were immunised four times subcutaneously with a dose of 10^8^ CFU of differentially inactivated *Brucella* species, either adjuvant-free or as adjuvanted formulation, in combination with the Sigma Adjuvant System® (SAS, Sigma Aldrich). Mice received either gamma (γ) irradiated *B. melitensis* in sterile Phosphate buffered saline (PBS, Sigma Aldrich), γ irradiated *B. melitensis* with SAS, formalin inactivated *B. melitensis* in PBS or formalin inactivated *B. abortus* in PBS.

Three days before cell fusion, two selected mice received an intravenous booster injection with 10^8^*Brucella* cells in PBS. Myeloma cells (PAI) were mixed 1:3 (fusion 1) and 1:1 (fusion 2) with spleen cells from the corresponding mouse in Iscove’s Modified Dulbecco’s Medium (IMDM, Sigma Aldrich). Cells were fused with 1 mL of pre-warmed (37 °C) Polyethylene glycol (PEG 800, Roche), dissolved in 150 mL HAT selective medium (IMDM 1 % 200 mM L-Glutamine (100X), 1 % Pen/Strep (100X, [+] 10,000 Units/mL Penicillin [+] 10,000 μg/mL Streptomycin, Gibco), 20 % FBS, HAT media supplement 50X Hybri-Max™, Sigma Aldrich) and cultured in 96-well tissue culture plates. Cells secreting *Brucella*-specific IgG were identified by ELISA coated with γ irradiated *B. melitensis* cells (16 M). From the two independent fusions, eleven hybridoma cell lines producing LPS specific mAbs were identified and cloned by limiting dilution. MAbs were purified from spent culture supernatant of the hybridoma clones by protein A affinity chromatography (HiTrap rProtein A FF, Amersham Biosciences). Purified mAbs were dialysed against PBS, aliquoted, and stored at −80 °C.

### Enzyme-linked immunosorbent assay (ELISA)

In indirect ELISA (iELISA), Maxisorp™ microtitre plates (Nunc, Thermo Scientific) were coated for 36 h at 4 °C with 50 μL of a 10 μg/mL solution of extracted LPS or with 50 μL of a bacterial suspension containing 10^7^ inactivated *Brucella* cells per mL. Wells were then blocked with 5 % milk powder in PBS for 2 h, followed by three washings with PBS containing 0.25 % Tween-20. Plates were incubated with appropriate dilutions of mouse sera or anti-LPS mAbs in PBS for 1–2 h at room temperature. After washing, plates were incubated with horseradish peroxidase-conjugated goat anti-mouse IgG (γ-chain specific) antibodies (Southern Biotech) for 1 h. TMB (TMB Microwell Peroxidase Substrate System (2-C), KPL) or ABTS substrate (ABTS® Peroxidase Substrate System, KPL) was added and incubated at room temperature until appropriate colour intensity was reached (five to 30 min). The optical density (OD) of the reaction product was recorded after 5 to 30 min at 570 nm or 405 nm using a microplate reader.

In antigen capture ELISA (cELISA), microtitre plates were coated with 50 μL of a 10 μg/mL solution of unlabelled mAbs in PBS. After being blocked and washed, wells were incubated with dilutions of inactivated *Brucella* cells in PBS. Biotinylated detection mAbs (10 μg/mL) were added and incubated for 1 h. After repeated washing, streptavidin-peroxidase polymer conjugate (1 μg/mL, Sigma Aldrich) was added and developed with the ABTS substrate.

Isotypes of anti-LPS mAbs were determined by detecting mAbs bound to anti-mouse lambda light chain antibody-coated plates with alkaline phosphatase-conjugated antibodies specific for mouse IgG1, IgG2a, IgG2b or IgG3 (Southern Biotech).

### Sodium dodecyl sulfate-polyacrylamide gel electrophoresis (SDS-PAGE) and immunoblotting

Aliquots of extracted LPS from *B. melitensis* and *B. abortus* were mixed with sample buffer (Laemmli buffer, Invitrogen) and heated for 15 min at 96 °C before loading on 4–12 % Bis-Tris gels. SeeBlue® pre-stained protein standard (Invitrogen) was used as a molecular weight marker. Following gel electrophoresis, LPS was transferred electrophoretically to nitrocellulose membranes. Blots were blocked for 2 h with 5 % milk powder in PBS, cut into strips and then incubated with purified mAbs (10 μg/mL) for 1 h. The strips were washed four times for 15 min with PBS containing 0.05 % Tween-20 and incubated with alkaline phosphatase-conjugated goat anti-mouse IgG heavy-chain antibodies (Sigma Aldrich) for 1 h. Strips were treated with 5-bromo-4-chloro-3-indolylphosphate and nitroblue tetrazolium to visualise bands.

### Immunofluorescence assay (IFA)

30 μL droplets of a fixing solution containing 4 % paraformaldehyde and 10 % PBS were placed in each well of a pre-coated Poly-L-Lysin microscope glass slide (Diagnostic Microscope Slides ES-242B-AD-CE24, Thermo Scientific). Ten μL of a bacterial suspension containing 10^8^ γ irradiated *B. melitensis* (16 M) or *B. abortus* (544) cells were added to each well and incubated for 30 min at room temperature. Wells were washed five times with PBS and then incubated for 15 min with 50 μL of blocking buffer containing 1 % fatty acid-free bovine serum albumin (BSA) in PBS. Afterwards, 30 μL of 10 μg/mL mAbs diluted in blocking buffer were added and incubated for 1 h. Wells were washed five times with blocking buffer before 30 μL of detection antibody (Alexa Fluor 568 (2 mg/mL, Invitrogen) conjugated donkey anti-mouse IgG (H + L), 1:400 in blocking buffer) was added for an additional hour. Finally, wells were washed five times, mounted with ProLong® Gold antifade reagent with 4',6-diamidino-2-phenylindole (DAPI, Invitrogen) and covered with a coverslip. Antibody binding and DNA staining were assessed by fluorescence microscopy.

### Luminex assay

Anti-LPS mAbs were coupled to magnetic beads (Bio-Plex Pro Magnetic COOH Beads, Biorad) according to the manufacturer's instructions and adjusted to a working concentration of 40 beads/μL in blocking buffer (1 % BSA in PBS). In the coupling reaction, 6 μg of antibody was applied to 5 × 10^5^ beads. Fifty μL of working bead mixture was used per microtitre well. Fifty μL bacterial samples were then added to each bead-containing well and incubated for 2 h on a microplate shaker at 37 °C in the dark. After incubation, the plates were washed with PBS containing 0.05 % Tween-20 and the beads were resuspended in 50 μL of biotinylated detection antibody at a concentration of 10 μg/mL in blocking buffer and incubated for 1 h. After repeated washing, 50 μL of a streptavidin-R phycoerythrin (ProZyme Inc.) solution was added and incubated for 30 min. The plate was then washed and the beads resuspended in 125 μL of blocking buffer before loading onto the BioPlex 200 instrument (Bio-Rad Laboratories). Reporter fluorescence was measured and expressed as mean fluorescence intensity of at least 100 beads per region. Multiplexed assays were performed in a single well format with mAb pairs 3D12/10G1 (*Brucellae*), MTA1/MTD6 [19], YPF19/YPF19 [22] and T14/FB11 [23].

### Statistical analysis

All data were obtained from experiments performed in duplicate (at a minimum). Antigen-free controls consisted of PBS (instead of sample suspended in PBS) and were further diluted with the diluent used for the particular assay. These controls were included in each experiment to determine the cut-off. Mean value, standard deviation and LOD (limit of detection) were calculated in Excel. Figure assembly, data transformation and non-linear regression (sigmoidal curve, dose–response variable slope) were done with GraphPad Prism.

## Results

### Generation and characterisation of *Brucella* LPS-specific mAbs

**Fig. 1:**
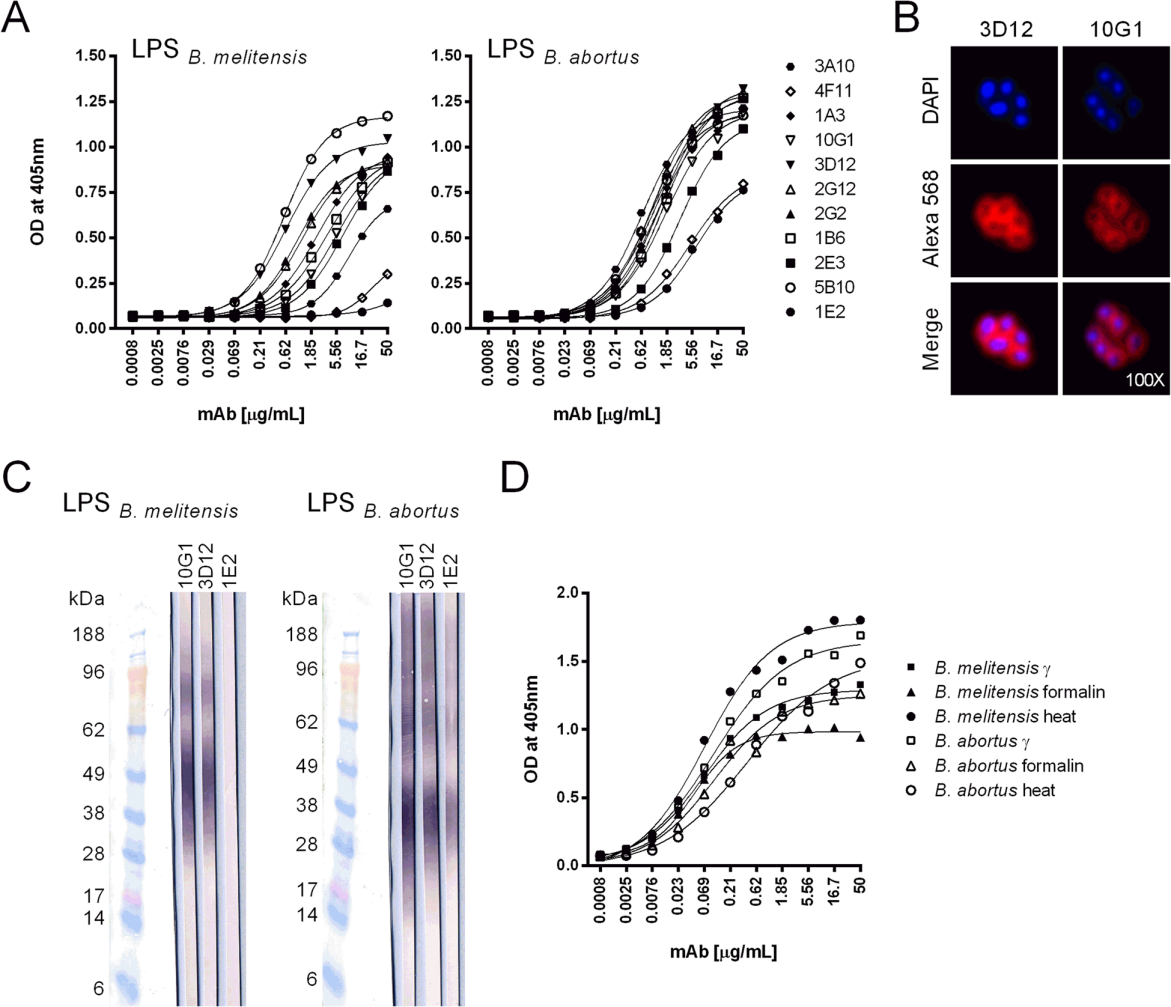
Antigen-binding properties of the generated *Brucella* LPS-specific mAbs. **a** Reactivity of the produced mAbs with extracted *B. melitensis* or *B. abortus* LPS in ELISA. **b** Western blot staining-patterns obtained with mAbs 10G1, 3D12 and 1E2 after SDS-PAGE of extracted *B. melitensis* and *B. abortus* LPS. **c** Indirect immunofluorescent staining of inactivated *B. melitensis* (16 M) cells by mAbs 3D12 and 10G1. The upper panel shows DNA staining with DAPI, the middle panel Alexa 568-specific immunofluorescence staining and the lower panel merged pictures of both stainings. **d** Reactivity of mAb 3D12 with gamma, formalin and heat inactivated *B. melitensis* (16 M) and *B. abortus* (544) cells in ELISA

Two mice exhibiting high ELISA IgG titres against *B. melitensis* (16 M) or *B. abortus* (NCTC 10093 544) cells after immunisation with inactivated bacterial cells were chosen for the generation of *Brucella* LPS-specific mAbs. Eleven hybridoma cell clones were obtained by screening with a *B. melitensis* (16 M, γ irradiated, 5 × 10^7^ CFU/mL) whole cell ELISA. Two mAbs (3A10 and 4 F11) were generated from a mouse immunised with γ irradiated *B. melitensis*, and nine (1A3, 10G1, 3D12, 2G12, 2G2, 1B6, 2E3, 5B10, 1E2) from a mouse immunised with formalin inactivated *B. abortus* cells. Determination of the mouse IgG subclass of the produced LPS-specific mAbs showed a predominance of the IgG2b(λ) isotype; only mAbs 4 F11 and 1E2 were of the IgG3(λ) isotype. While all 11 mAbs recognised extracted LPS from *B. abortus* (type A O-antigen), mAbs 1E2 and 4 F11 showed a markedly weaker reactivity with *B. melitensis* LPS (type M O-antigen) than did the others in ELISA (Fig. 1a) and Western blotting (Fig. 1c). The Western blot profiles (Fig. 1c) were typical for ‘smooth’ LPS of *Brucella sp.* [24]. In immunofluorescence analysis with inactivated *B. melitensis* and *B. abortus* cells, all anti-LPS mAbs yielded a homogenous circular surface staining. Figure 1b shows a representative staining for mAbs 3D12 and 10G1 with *B. melitensis* cells. The differences in the fine-specificities of the mAbs observed in ELISA correlated with differences in immunofluorescence analysis, where surface staining by mAbs 1E2 and 4 F11 with *B. melitensis* cells was weak (data not shown). For the analysis of the samples under biosafety level two conditions, inactivation is required. Different methods, γ irradiation, formalin inactivation and heat treatment, are available for that. Irrespective of the inactivation method, the anti-LPS mAbs reacted with *B. melitensis* and *B. abortus* cells in ELISA (Fig. 1d).

**Fig. 2:**
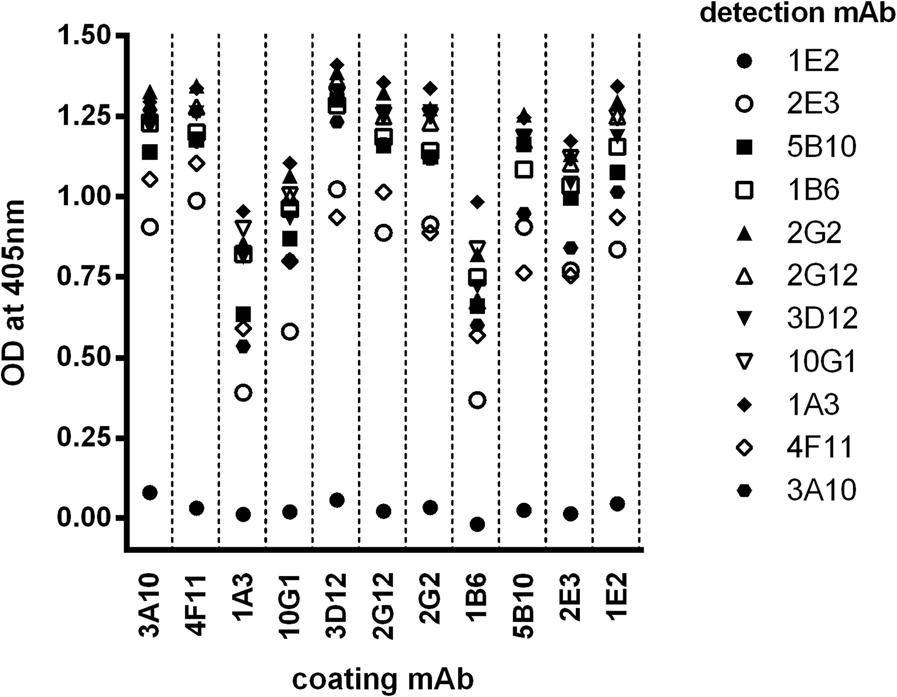
Comparative testing of mAb pairs in an antigen capture ELISA. To evaluate optimal antibody combinations, each of the 11 mAbs was used as a capture or detection (biotin-conjugate) antibody at a concentration of 10 μg/mL. Optical densities were measured for each antibody combination using gamma-irradiated *B. melitensis* (16 M) cells at a concentration of 10^7^ cells/mL

To develop a highly sensitive antigen capture assay, a suitable combination of a capturing and a biotinylated-detecting mAb was selected from the pool of 11 mAbs. In a sandwich ELISA format, the majority of mAb combinations tested were suitable for detecting *B. melitensis* cells (Fig. 2). Despite its weak reactivity with *B. melitensis* LPS, mAb 1E2 could effectively be used as a capture antibody but it failed to interact with *B. melitensis* cells when used as a detection antibody. A differentiation between *B. melitensis* and *B. abortus* cells was thus only observed with mAb 1E2 as a detection antibody (data not shown). MAb 3D12 performed best as an antigen capture antibody while mAb 10G1 was selected as the detection antibody as it gave the highest read out in combination with mAb 3D12 as the capture antibody. Hence, further development focussed on the mAb pair 3D12/10G1.

### Developing a Luminex assay for rapid and sensitive detection of *Brucella spp.*

**Fig. 3:**
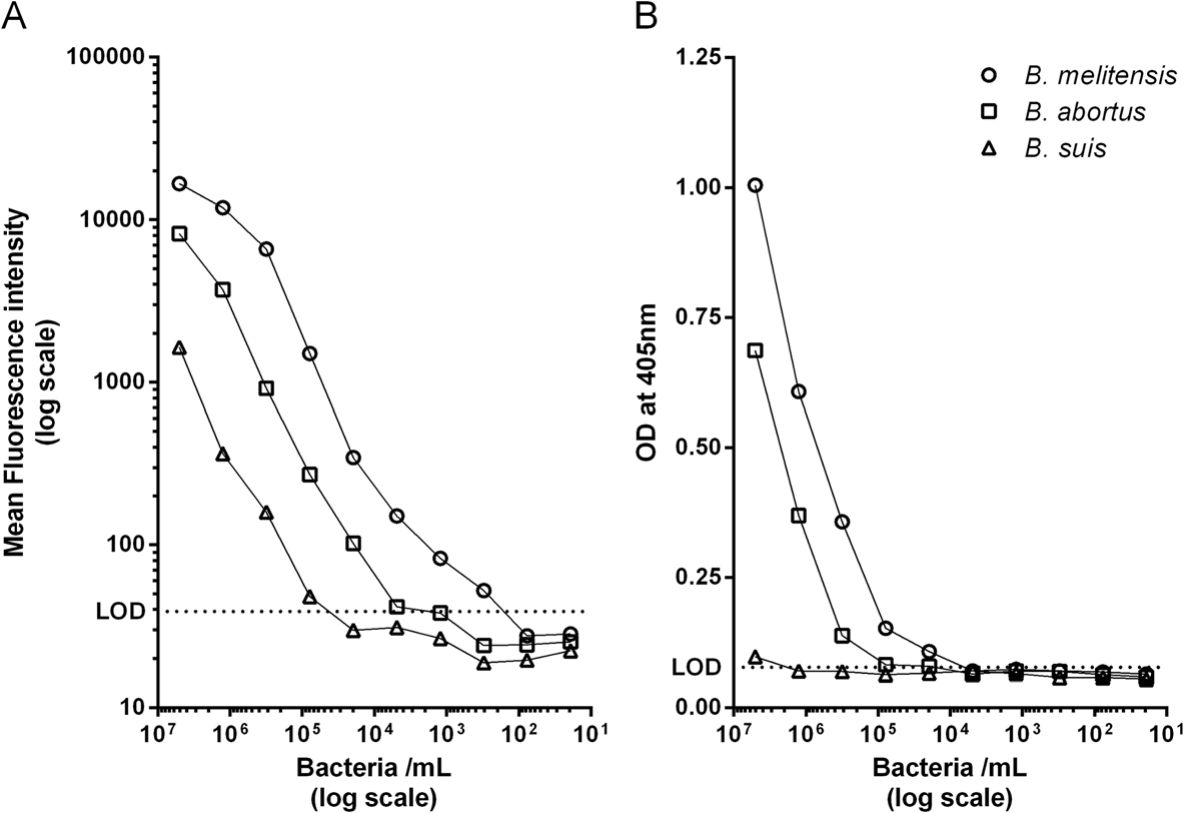
Comparison of the sensitivity of the bead-based Luminex immunoassay (**a**) and the corresponding antigen capture ELISA (**b**). Assay sensitivities were determined by analysing serial dilutions of inactivated *B. melitensis* (16 M, γ), *B. abortus* (544, γ) and *B. suis* (1330, formalin) cells. Dashed lines indicate the assay dependent limit of detection (LOD) defined as mean blank (i.e. the no-antigen control) plus three times the standard deviation (SD)

The mAb pair 3D12/10G1 was used to develop an antigen capture assay based on Luminex xMAP technology. While similar to ELISA in overall assay format, the Luminex technology combines advanced fluidics, optics, and digital signal processing with up to 500 color-coded microspheres to provide an accurate measurement of multiple analytes from a single sample [25]. Each bead set can be conjugated to a specific biomolecule (such as an antibody) to capture analytes of interest using a very small sample volume. Here, the mAb 3D12 was coupled to magnetic beads and used as the capture antibody, and the biotinylated mAb 10G1 was used as the detection antibody. The sensitivity of this Luminex assay was determined by analysing serial dilutions of inactivated *B. melitensis*, *B. abortus* and *B. suis* cells. The limit of detection (LOD) was calculated as the mean fluorescence intensity of the blank plus three times the standard deviation (SD) and set as the threshold (dashed line in Fig. 3a). The detection limits in a sample volume of 50 μL were 2 × 10^2^ cells per mL for *B. melitensis*, 5 × 10^3^ cells per mL for *B. abortus* and 8 × 10^4^ cells per mL for *B. suis*. Depending on the species tested, the sensitivity of the Luminex assay was 4 to 50 times higher than that of a corresponding antigen capture ELISA (Fig. 3b), where at least 10^4^, 2 × 10^4^ and 3 × 10^6^ cells per mL, respectively, were required for accurate detection.

The specificity of the bead-based assay was tested with several biotypes of *B. melitensis* (1–3), *B. abortus* (1,3), *B. suis* (1,2), *B. canis, B. ovis and B. neotomae,* as well as with other potential bioterror agents (*F. tularensis, B. anthracis, S. typhimurium, Y. pestis, B. mallei, B. pseudomallei* [1]) and bacteria (*Y. enterocolitica O9, E. coli O157* and *V. cholera O1* [26–29]) with structurally similar O-antigens of α1,2-linked 4-amino-4,6-dideoxy-α-D-mannopyranosyl subunits and *O. anthropi*, the closest relative of *Brucellae* [30]. The Luminex assay detected all ‘smooth’ *Brucella* species (*B. melitensis, B. abortus, B. suis* and *B. neotomae*) independently of their biotype (Table 2). Overall, *Brucella* species expressing the M O-antigen were detected with higher sensitivity compared to A or AM O-antigen expressing *Brucella*. Cross-reactivity with *Y. enterocolitica O9* was found, as predicted by the structural identity of the type A O-antigen [27, 28]. Neither *B. canis* nor *B. ovis* cells expressing a ‘rough’ LPS nor any of the other bacterial species tested gave positive signals.

**Table 2:** Specificity of the developed bead-based Luminex immunoassay

Bacterial species	Strain	O-Antigen	Luminex assay (mean fluorescence intensity)
*Brucella spp. (biotype)*			
* B. abortus (1)*	NCTC 10093 544	A^1,3^	**13107**
	19S	A^1,3^	**11300**
	A146-10	A^1,3^	**5607**
* B. abortus (3)*	A104-10 Uckermark	A^1,3^	**3172**
* B. canis*	NCTC 10854 RM-666	R^2,3^	33
* B. ovis*	CNCTC 6741	R^2,3^	23
* B. melitensis (1)*	NCTC 10094 16 M	M^1,3^	**25860**
	ICM 3.33	M^1,3^	**13129**
	ICM 583/2003	M^1,3^	**16972**
	ICM 91/2004	M^1,3^	**15030**
	102A01C2F	M^1,3^	**22285**
	A146-13	M^1,3^	**11407**
	A104-11 Tgb. Nr. 117518	M^1,3^	**5977**
* B. melitensis (2)*	A104-12 799/97, B3898	A^1,3^	**2958**
* B. melitensis (3)*	A104-13 210739, Mainz	AM^1,3^	**1884**
* B. suis (1)*	NCTC 10316 1330	A^1,3^	**5167**
* B. suis (2)*	A 104–14 Rostock	A^1,3^	**10316**
* B. neotomae*	A148-7 5 K33	A^1,3^	**1875**
Other bacteria			
* F. tularensis tularensis*	ATCC 6223	D^4^	18
* F. tularensis holarctica*	NCTC 10857	D^4^	22
* B. anthracis (spores)*	Böhm 73202.2000 (PX02)	D^5^	19
* S. typhimurium*	ATCC 14028	D^6^	19
* Y. enterocolitica*	310 (IT2, ST9) O9	A^7,8,9^	**1291**
* Y. pestis*	CO92	R^10^	20
* O. anthropi*	ATCC 49188	D^11^	20
* E. coli*	O157, 15326	A^7,12^	22
* B. mallei*	NCTC 03709 106	D^13^	18
* B. pseudomallei*	H05410-0490	D^14^	20
* V. cholera*	O1, ATCC 14734	A^15^	24

Luminex LOD was defined as two times the mean fluorescence intensity of the blank (mean blank = 20) and used as the threshold for positive results. Values in bold indicate positive results. Classification of O-antigens [4, 26–29, 41–50]: A = α1,2-linked 4-amino-4,6-dideoxy-α-D-mannopyranosyl subunits, M = α1,3-linked and α1,2-linked 4,6-dideoxy-4-formamido-α-D-mannopyranosyl residues, D = different O-antigen structure compared to *Brucella*, R = ‘rough’ LPS (no O-antigen). Meikle et al. 1989^1^, Adone et al. 2011^2^, Corbel 2006^3^, Wang et al. 2011^4^, Crich and Vinogradova 2007^5^, Watson et al. 1992^6^, Perry et al. 1986^7^, Caroff et al. 1984^8^, Bundle et al. 1984^9^, Skurnik et al. 2000^10^, Velasco et al. 1996^11^, Perry and Bundle 1990^12^, Burtnick et al. 2002^13^, Perry et al. 1995^14^, Kenne et al. 1982^15^

**Fig. 4:**
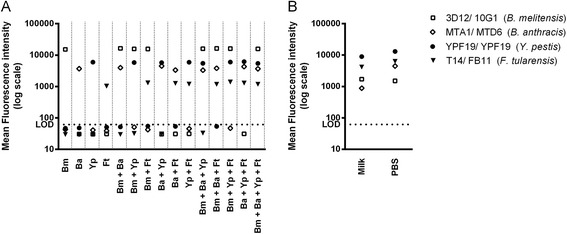
Multiplexed Luminex immunoassay for detecting potential bioterror agents, *B. melitensis*, *B. anthracis*, *F. tularensis* and *Y. pestis*. **a** Test samples contained *B. melitensis* 16 M (Bm, 5 × 10^5^ cells/mL), *B. anthracis* PXO1+ (Ba, 5 × 10^5^ cells/mL), *F. tularensis* 6223 (Ft, 5 × 10^5^ cells/mL) and *Y. pestis* CO92 (Yp, 5 × 10^4^ cells/mL) cells in PBS either alone or in combination. In (**b**), PBS and milk samples were spiked with all four bacterial species and used at a concentration of 2.5 × 10^6^ cells/mL. MAbs 3D12, MTA1, T14 and YPF19, coupled to distinct magnetic beads, were used as capture antibodies and the biotinylated mAbs 10G1, MTD6, FB11 and YPF19 were used for detection. Reporter dye fluorescence intensities measured for each bead set are shown. Dashed lines indicate the limit of detection (LOD) defined as mean blank plus three times the standard deviation

The newly developed singleplex assay for *Brucella spp.* was integrated into a previously established multiplex assay to allow for simultaneous detection of the four potential bioterror agents, *B. melitensis, B. anthracis*, *F. tularensis* and *Y. pestis*, in a single run of the assay. Mixed samples containing combinations of the four bacterial species were prepared in PBS and tested in the multiplexed immunoassay format (Fig. 4a). All four bio-threat agents tested were accurately detected and no cross-reactivities between individual singleplex assays were observed. The specificity of the Luminex assay for *B. anthracis, F. tularensis* and *Y. pestis* had been tested prior to the multiplex testing (Additional files 1 and 2).

In addition, the multiplex assay specifically identified all four bacterial species from a spiked milk sample, indicating that the newly developed Luminex assay is also suitable for detecting *Brucella spp.* in complex biological samples (Fig. 4b).

## Discussion

Brucellosis is one of the most common bacterial zoonosis worldwide and an important cause of economic losses and human suffering [2, 4]. Moreover, *B. abortus*, *B. melitensis* and *B. suis* could be developed as bioterrorism agents due to their ability to undergo aerosolization [31]. Isolation by cultivation is the standard method for identifying *Brucella* bacteria in biological samples, but may take up to 4 weeks to complete. Methods based on the polymerase chain reaction that identifies nucleic acid fragments from bacteria are becoming more practical for detecting *Brucella spp*. [32, 33]. However, according to international biodefense regulations, immunological detection methods for potential bioterror agents are required in addition to molecular detection and identification assays.

In this study, we showed that *Brucella* O-antigen-specifc mAbs represent potent immuno-capturing components for a highly sensitive detection system for *Brucella* cells in complex samples. Immunisation of laboratory mice with inactivated *Brucella* bacteria combined with a *B. melitensis* whole cell ELISA for selecting B-cell hybridoma lines that produce *Brucella*-specific antibodies yielded exclusively LPS-specific mAbs, although anti-protein IgG antibodies could also be detected in the serum of the immunised mice (data not shown). This observation might be explained by the fact that in ‘smooth’ *Brucella* species, outer membrane proteins and other membrane components are masked by O-polysaccharide chains of LPS [34, 35]. All of the mAbs generated recognised LPS from *B. abortus* (type A O-antigen) and from *B. melitensis* (type M O-antigen). However, mAbs 1E2 and 4 F11 differed from the other mAbs in fine-specificity in that they showed a markedly reduced reactivity with *B. melitensis* LPS. None of the sample inactivation methods tested (gamma irradiation, formalin and heat treatment) affected the interaction between the mAbs produced and the bacterial cells, corroborating the suitability of *Brucella* LPS as a stable target antigen for detection. Dependent on infrastructural constraints (i.e. availability of gamma irradiation) and application, laboratories may have different preferences concerning the inactivation method.

As expected from the comparative binding studies, the majority of the mAb combinations tested were suitable for detecting *Brucella* cells in a sandwich capture ELISA format. A differentiation between *B. melitensis* and other *Brucella* species expressing ‘smooth’ LPS was achieved with mAb 1E2 in a suitable test format. The Luminex immunoassay with the selected mAb pair, 3D12 and 10G1, captured and detected cells of all ‘smooth’ *Brucella* species and biotypes tested but also showed cross-reactivity with *Y. enterocolitica* serotype O9. The-O-antigens of *Y. enterocolitica O9, E. coli O157, V. cholera O1* and *B. abortus* all consist of a linear polymer of α1,2-linked 4-amino-4,6-dideoxy-α-D-mannopyranosyl residues (perosamine). However, they differ in the *N*-acylation of the perosamine sugar [26]. While *B. abortus* and *Y. enterocolitica O9* are N-acylated with formic acid, *V. cholera O1* are substituted with (S)-2,4-dihydroxybutanoic acid [36]. These derivatisations can have major effects on antibody binding, which may explain why our mAbs only showed cross-reactivity with *Y. enterocolitica O9*. PCR can verify whether a result obtained with mAb pair 3D12/ 10D1 is true or false positive due to *Y. enterocolitica O9* contamination. To conclusively analyse environmental samples a combination of molecular and immunological methods is recommended [37]. Our mAbs are specific to *Brucella* carrying ‘smooth’ LPS, hence a detection of ‘rough’ *Brucella* species is not possible. A LPS-independent detection based on surface-exposed structures might solve this problem.

Depending on the *Brucella* species tested, the assay was able to detect 10 to 4000 cells in a sample volume of 50 μL. Currently available molecular identification assays for *Brucella spp*. offer comparable or even lower detection limits [32, 33, 38].

Recently, a capture ELISA for diagnostic purposes was developed using LPS-specific monoclonal antibodies to detect LPS antigens in the blood [39]. Both, our approach for generating LPS-specific monoclonal antibodies, as well as the overall purpose of our test development were different. We developed a highly sensitive Luminex multiplex assay for the detection of biological threat agents both in natural outbreak and bio-threat situations.

The conversion of the ELISA into the Luminex bead-based assay markedly increased the sensitivity for detecting *Brucella* and allowed integration of the *Brucella* assay into a multiplex assay to simultaneously detect a range of relevant bio-threat species. The multiplexed immunodetection assay accurately detected *Brucella spp*., *B. anthracis*, *F. tularensis* and *Y. pestis* cells within a single mixed sample. Brucellosis is transmitted to humans through consumption of unpasteurised dairy products or through direct contact with infected animals. Although detecting *Brucella* cells in milk is complicated [40], the Luminex multiplex assay specifically identified all tested bacterial species from spiked milk samples, demonstrating that the developed assay is a suitable tool for detecting *Brucella* cells in complex samples.

## Conclusion

The Luminex assay described here is a suitable tool for specifically detecting *Brucella spp*. even in complex samples such as milk. Four bio-threat agents can be detected in the multiplex format, quickly and specifically. Overall, using the Luminex assay together with common molecular and cultivation methods is crucial to fulfilling international biodefense regulations for rapidly and reliably identifying biological threat agents. In the future, the Luminex assay may also be considered for detecting *Brucella* in clinical samples.

## Additional files

Additional file 1:
**Additional bacterial strains (** ***Bacillus, Yersinia, Francisella*** **) included in the study.** (PDF 279 kb) Additional file 2:
**Specificity of the developed bead-based Luminex immunoassay for** ***B. anthracis, F. tularensis*** **and** ***Y. pestis.*** (PDF 170 kb)
