# Supplementary material for: Development of a bead-based Luminex assay using lipopolysaccharide specific monoclonal antibodies to detect biological threats from Brucella species
Source: BMC Microbiol. 2015 Oct 5;15:198. doi: 10.1186/s12866-015-0534-1 (PMC4595103; doi:10.1186/s12866-015-0534-1)
Supplement: Additional file 1: — Additional bacterial strains ( Bacillus, Yersinia, Francisella ) included in the study. (PDF 279 kb) [file 12866_2015_534_MOESM1_ESM.pdf]

Additional file 1: Additional bacterial strains (*Bacillus, Yersinia, Francisella*) included in the study

Bacterial species	Strain/Isolate	Inactivation method	Source
Bacillus spp. spores			
B. anthracis	ames	ɣ-irradiated	USCRP
	Böhm A58	10% formaldehyde	IVB,B
	Böhm A1	10% formaldehyde	IVB,B
	Böhm A 73202.2000	10% formaldehyde	IVB,B
	NCTC 8234	10% formaldehyde	NCTC
	NCTC 10340	10% formaldehyde	NCTC
	NCTC 01328	10% formaldehyde	NCTC
	NCTC 02620	10% formaldehyde	NCTC
	W514 Bern	10% formaldehyde	IVB,B
B. cereus	ATCC10876	10% formaldehyde	ATCC
	ATCC11778	10% formaldehyde	ATCC
	ATCC13061	10% formaldehyde	ATCC
	ATCC14579	10% formaldehyde	ATCC
	ATCC33019	10% formaldehyde	ATCC
B. subtilis	ATCC6051	none	ATCC
	ATCC6633	none	ATCC
	ATCC11774	none	ATCC
B. thuringiensis ssp. kurstaki	SP09	10% formaldehyde	SIBA
	ATCC10792	10% formaldehyde	ATCC
B. athropheaeus	ATCC9372	none	ATCC
B. circulans	ATCC61	none	ATCC
B. licheniformis	ATCC12759	none	ATCC
	ATCC14580	none	ATCC
B. megaterium	ATCC9885	none	ATCC
	ATCC14581	none	ATCC

B. sphaericus	ATCC4525	none	ATCC
B. pumilus	ATCC14884	none	ATCC
Yersinia spp. (biotype)			
Y. pestis	ICM 1/41	3%formalin	ICM
	ICM 1/48	3%formalin	ICM
	EV 76	3%formalin	RKI
	CO92	3%formalin	DRDC
	NCTC (0)2868	3%formalin	NCTC
	NCTC 10030 (13927/58)	3%formalin	NCTC
Y. pseudotuberculosis	BV1, cattle	3%formalin	ICM
Y. pseudotuberculosis	BV2, cattle	3%formalin	ICM
Y. pseudotuberculosis	BV3, cattle	3%formalin	ICM
Y. pseudotuberculosis	BV4, cattle	3%formalin	ICM
Y. pseudotuberculosis	1.5, Lausanne 2931	3%formalin	ICM
Y. enterocolytica (1)	309, human	3%formalin	ICM
Y. enterocolytica (2)	382, human	3%formalin	ICM
Y. enterocolytica (3)	371, human	3%formalin	ICM
Y. enterocolytica (4)	28, water	3%formalin	ICM
Francisella spp.			
F. philomiragia	ATCC25015	3% formalin	ATCC
F. tularensis ssp. tularensis	ATCC 6223	3% formalin	IVB,Z
	Schu 4	3% formalin	RKI
F. tularensis ssp. holarctica	NCTC 10857	3% formalin	NCTC
	JF 3824, human	3% formalin	IVB,B
	JF 3825, monkey	3% formalin	IVB,B
	human, (Ft5)	3% formalin	KSBL
	human, (Ft6)	3% formalin	KSA
	human, (Ft7)	3% formalin	KSA
	human, (Ft8)	3% formalin	KSA
	human, (Ft9)	3% formalin	KSA
	JF 3820, hare	3% formalin	IVB,B
	JF 3821, hare	3% formalin	IVB,B

	JF 3822, hare	3% formalin	IVB,B
	JF 3826, monkey	3% formalin	IVB,B
	JF 3829, monkey	3% formalin	IVB,B
	JF 3859, hare	3% formalin	IVB,B
	JF 4092, hare	3% formalin	IVB,B
	JF 4128, human	3% formalin	IVB,B
	JF 4212, human	3% formalin	IVB,B
	JF 4242, hare	3% formalin	IVB,B
	JF 4429, human	3% formalin	IVB,B
	JF 4455, hare	3% formalin	IVB,B
	JF 4456, human	3% formalin	IVB,B

Bacteria were inactivated by 3% formalin, 10% formaldehyde or not. NCTC = National Collection of Type Cultures (London, UK), ICM = Instituto cantonale die microbiologica, Bellinzona (Bellinzona, Switzerland), DRDC = Defence Research and Development Canada (Ottawa, Canada), IVB,Z = Institut für Veterinärbakteriologie der Uni Zürich (Zurich, Switzerland), IVB,B = Institut für Veterinärbakteriologie der Universität Bern (Bern, Switzerland), KSBL = Kantonsspital Liestal (Liestal, Switzerland), KSA = Kantonsspital Aarau (Aarau, Switzerland), USCRP = U.S. Critical Reagents Program, SIBA = Sampling and Identification of Biological Agents, Isolate from Profiency Test 2009, RKI = Robert Koch Institute, Isolates from Profiency Tests (EQADeBa, QUANDHIP, Berlin, Germany), ATCC = American Type Culture Collection.
