# Supplementary material for: Development of a bead-based Luminex assay using lipopolysaccharide specific monoclonal antibodies to detect biological threats from Brucella species
Source: BMC Microbiol. 2015 Oct 5;15:198. doi: 10.1186/s12866-015-0534-1 (PMC4595103; doi:10.1186/s12866-015-0534-1)
Supplement: Additional file 2: — Specificity of the developed bead-based Luminex immunoassay for B. anthracis, F. tularensis and Y. pestis. (PDF 170 kb) [file 12866_2015_534_MOESM2_ESM.pdf]

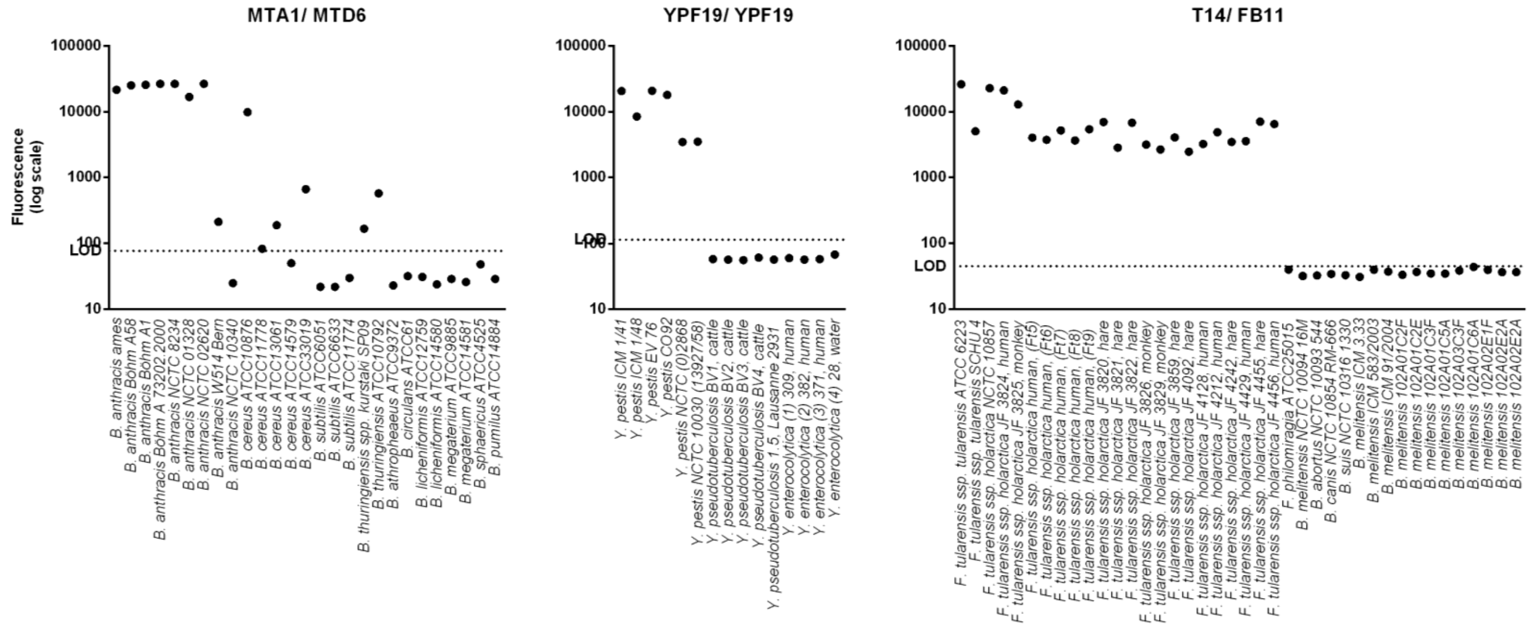

Additional file 2: Specificity of the developed bead-based Luminex immunoassay for *B. anthracis, F. tularensis* and *Y. pestis*. MAb pairs MTA1/ MTD6 [19], YPF19/ YPF19 [22] and T14/ FB11 [23] were analysed for their specific detection of *B. anthracis*, *Y. pestis* and *F. tularensis*, respectively. Coating mAbs (6μg) were coupled to $5x10^5$ magnetic beads (Bio-Plex Pro Magnetic COOH Beads, Biorad). The working concentration was 40 beads/μL in blocking buffer (1% BSA in PBS) with 50μL per well. Each bead containing well was incubated for 2 hours with 50μL bacterial samples (*Bacillus* and *Francisella spp*., $5x10^6$ cells/ml, *Yersinia spp*., $5x10^5$ cells/mL) on a microplate shaker at 37°C in the dark. Plates were washed with PBS containing 0.05% Tween-20 and the beads incubated in 50μL of biotinylated detection antibody (10μg/mL in blocking buffer) for 1 hour. After repeated washing, 50μL of a streptavidin-R phycoerythrin (ProZyme Inc.) solution was added and incubated for 30 min. The plate was washed and the beads resuspended in 125μL of blocking buffer before loading onto the BioPlex 200 instrument (Bio-Rad Laboratories). Reporter fluorescence was measured and expressed as mean fluorescence intensity of at least 100 beads per

region. Dashed lines indicate the assay dependent limit of detection (LOD) defined as mean blank (i.e., the no-antigen control) plus three times the standard deviation (SD). Data were collected from three independent experiments in duplicates.
